# Top Five Infectious Diseases Card Games for Medical Learners

**DOI:** 10.7759/cureus.113860

**Published:** 2026-08-03

**Authors:** Saira Butt, Nabiha Sohail

**Affiliations:** 1 Internal Medicine/Infectious Diseases, Indiana University School of Medicine, Indianapolis, USA; 2 Internal Medicine, Fatima Memorial Hospital (FMH) College of Medicine and Dentistry, Lahore, PAK

**Keywords:** card game, empiric, fellows, gamification, infectious diseases, medical education, medical students, pharmacology, pharmd, residents

## Abstract

Educational card games are increasingly used to teach microbiology, infectious disease management, and antimicrobial stewardship across the medical education continuum, from undergraduate coursework through residency and fellowship. This technical report identifies and evaluates infectious-diseases-themed card games (excluding board games) and ranks them based on their suitability for medical students, pharmacy students, residents, and fellows. Games were identified through targeted web searches, retailer searches, publisher and crowdfunding-platform listings, and a search of the peer-reviewed medical education literature, including PubMed, MedEdPORTAL, and the Journal of Microbiology and Biology Education. Five games met the inclusion criteria and are profiled in depth. Games ranged from free, peer-reviewed, guideline-derived instructional tools to commercially available retail products. Ranked from most to least suited to medical learners, the games are Empiric, Pharmageddon: Bugs vs Drugs, Pathogen Pursuit, Epipocalypse: Bug Detectives, and Healing Blade: Defenders of Soma. Ranking criteria included clinical and guideline fidelity, emphasis on antimicrobial stewardship, evidence of learning benefit, and practicality of use, including cost, availability, and solo versus facilitated play. Free, guideline-anchored, peer-reviewed games offer the strongest fit for clinical learners, while commercially polished products trade some clinical depth for production value and broader appeal.

## Introduction

Educational card games have become an increasingly popular tool for teaching microbiology, infectious disease epidemiology, and antimicrobial stewardship to audiences ranging from grade-school science classes to medical residency programs. Compared with lectures or flashcards, card games introduce competition, peer discussion, and scenario-based reasoning, which published studies associate with improved knowledge retention and learner engagement [1]. This technical report profiles five notable infectious-disease-themed card games designed specifically for medical learners. Games pitched to general audiences or undergraduate biology learners were excluded from evaluation. Each profile summarizes how the game is played, its learning objectives, its intended audience, cost, availability, ease of use, popularity, and a balanced evaluation of its strengths and weaknesses. The games were ranked from most to least suitable for medical students, pharmacy students, residents, and fellows, with the comprehensive rationale detailed in the discussion section.

Prior systematic and scoping reviews of game-based learning in health professions education report generally positive but heterogeneous effects on knowledge and engagement, and several authors have called for more rigorous, comparative evaluation of individual products rather than the game-based approach in general [2,3]. The rationale for card games specifically draws on established educational theory, including active learning [4], experiential learning (learning through structured practice and reflection rather than passive instruction) [5], and retrieval practice and spaced repetition (repeated, effortful recall of information over time, which is associated with improved long-term retention) [6]. Despite the proliferation of infectious-disease-themed card games marketed to health professions learners, no prior report has systematically identified and compared the games currently available to this specific audience or evaluated their fit against clinical guideline fidelity and antimicrobial stewardship principles; this technical report addresses that gap.

## Technical report

Candidate games were identified through a structured search conducted in July 2026. The search utilized general web search engines, combining terms such as "infectious disease card game", "antibiotic card game", "microbiology card game education", and "medical education card game". Additionally, peer-reviewed medical and science education literature databases, including PubMed, MedEdPORTAL, and the Journal of Microbiology and Biology Education, were searched to capture games described in published, peer-reviewed studies with reported learning outcomes. Publisher and manufacturer websites, such as Genius Games, Nerdcore Medical, Lab Beagle Games, and the Sanford Guide, were reviewed for authoritative descriptions of game mechanics, components, and pricing. Retail and marketplace listings, such as Amazon, Target, Etsy, Boardlandia, By The Board Games, Continuum Games, and Rainbow Resource, were checked to confirm current pricing and availability. Crowdfunding platforms, including Kickstarter and Gamefound, were searched for games that originated from or remain available through crowdfunded campaigns. At the same time, community and hobbyist references such as BoardGameGeek, Student Doctor Network forums, and GeekDad were consulted to corroborate gameplay descriptions and gauge community reception.

Inclusion criteria required that the game be card-based rather than board-based and that its primary subject matter cover an infectious disease theme, pathogen, antimicrobial, or related epidemiological and microbiological concept. Eligible games were required to have sufficient publicly available information describing gameplay, learning objectives, cost, and availability. Exclusion criteria included traditional board games, games not oriented toward clinical or health-professional education, and games for which insufficient public information was available. Because this report's specific aim is to evaluate fit for medical students, pharmacy students, residents, and fellows, two games identified in an earlier and broader search, Virulence: An Infectious Card Game and the Pathogenesis Card Game, were excluded because their target audiences fall outside this professional population. Five games met all inclusion criteria and were evaluated.

**Figure 1:**
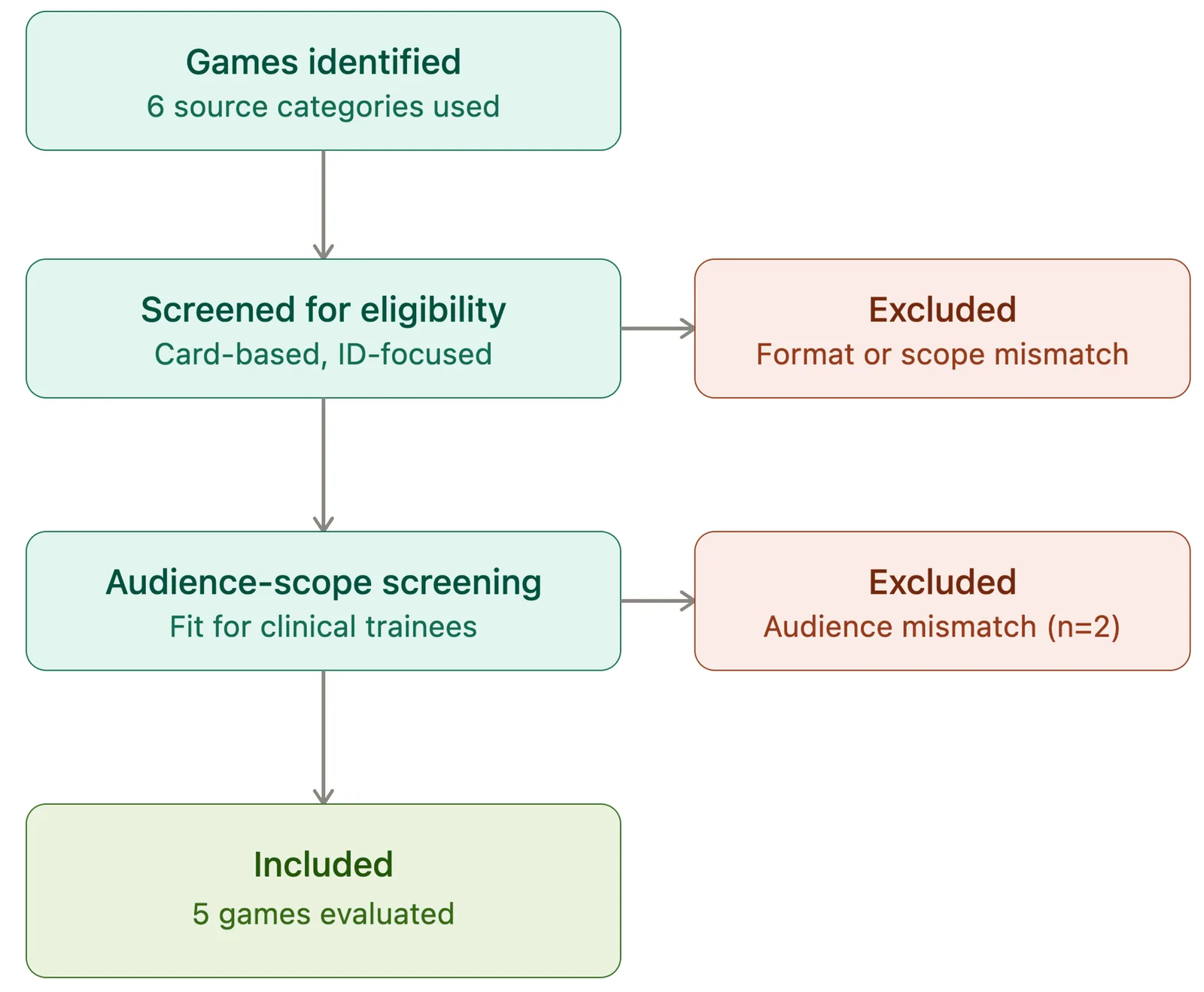
Flow diagram of game identification and selection Because the search strategy was exploratory rather than conducted using a predefined, logged, stage-based protocol, stage-specific record counts are unavailable and therefore not presented. Instead, the diagram illustrates the categories of games considered and the inclusion and exclusion criteria applied during the selection process, rather than quantitative PRISMA-style screening counts. PRISMA: Preferred Reporting Items for Systematic Reviews and Meta-Analyses

This report is best characterized as a structured, qualitative expert evaluation rather than a systematic review. Consistent with that scope, the search was iterative and exploratory rather than governed by a fixed, reproducible protocol: exact search strings, search dates for each database and platform, and a Preferred Reporting Items for Systematic Reviews and Meta-Analyses (PRISMA)-style flow diagram of records identified, screened, and excluded were not systematically logged and are therefore not reported (Figure 1). No standardized, validated scoring instrument was used to rank the games; instead, the authors applied the four weighted considerations described below as a qualitative framework, and the resulting rankings reflect informed expert judgment rather than statistically validated or independently reproducible measurements. Readers should interpret the rankings accordingly.

The qualitative, narrative ranking of the five included games, from most to least suitable for medical learners, was performed by the authors of this technical report, based on a structured evaluation of four weighted considerations. These considerations included clinical and guideline fidelity, explicit emphasis on antimicrobial stewardship concepts rather than simple pathogen-drug recall, availability of published or reported evidence of learning benefit, and practicality for this population, including cost, solo usability, and current availability. Table 1 summarizes the format, cost, and availability of five infectious disease card games.

**Table 1:** Summary of format, cost, and availability, ranked best to worst fit for medical learners

Rank	Game	Format/length	Cost	Availability
1	Empiric (Sanford Guide Edition)	2-4 players, 20-30 min	Free (print-and-play)	Free download via empiricgame.com
2	Pharmageddon: Bugs vs Drugs	1-4 players, 20-60 min	~$30-$40	Kickstarter/Gamefound backers; Etsy, Amazon
3	Pathogen Pursuit	Group/team format, 60-120 min	Free (institution-run)	Published protocol via MedEdPORTAL
4	Epipocalypse: Bug Detectives	1-4 players, ~20-30 min	Free (print-and-play)/TBD retail	Free download; Kickstarter planned
5	Healing Blade: Defenders of Soma	2 players, ~30-45 min	~$60-$70	Retail (Nerdcore Medical store, Target, Amazon)

The highest-ranked game is Empiric (Sanford Guide Edition): Antibiotic Card Game, which is developed independently in partnership with the Sanford Guide [7,8]. In this game, players match antibiotic cards to clinical case cards, scoring more points for guideline-concordant, narrow-spectrum choices than for inappropriate broad-spectrum or off-target selections. Held cards visually assemble into a condensed antimicrobial spectrum-of-activity table, reinforcing pattern recognition for two to four players. The learning objectives are to apply guideline-based antibiotic selection in common clinical scenarios, reinforce antimicrobial stewardship principles by rewarding guideline-adherent prescribing, and build a mental spectrum-of-activity map. The target audience includes physicians, pharmacists, nurse practitioners, and veterinary professionals in training, and the game is free to download as a print-and-play PDF. It is considered moderately to highly challenging, and its strengths include being free, guideline-derived, and validated in published educational research [9]. However, its print-and-play format lacks physical durability, and its high level of difficulty may frustrate beginners without instructor guidance.

The second-ranked game is Pharmageddon: Bugs vs Drugs, published by Lab Beagle Games [10,11]. In this game, players use drug cards to eliminate bug cards laid out on a shared grid, with each drug consuming health and gut flora resources to discourage broad-spectrum overuse. The game supports solo, cooperative, and versus modes across multiple difficulty levels for one to four players. Its learning objectives are to practice matching appropriate antibiotics to specific pathogens, understand the trade-offs involved in antimicrobial stewardship, and recognize complications in special populations. It is designed for medical, pharmacy, and nursing trainees and costs roughly $30-$40 [12,13]. While its strengths include solo-play capability and explicit teaching of stewardship trade-offs, its limitations include limited print runs and a lack of large-scale, peer-reviewed efficacy data.

The third-ranked game is Pathogen Pursuits, an academic game published as a protocol on MedEdPORTAL [14]. Participants earn antimicrobial cards by correctly answering infectious disease questions, then match those cards to pathogen cards representing organisms such as methicillin-resistant *Staphylococcus aureus*, *Enterococcus faecalis*, and *Mycobacterium avium* complex. Sessions run 60 to 120 minutes and are facilitated group activities. The learning objectives are to select appropriate antimicrobial treatments, reinforce stewardship principles, and improve knowledge retention, as demonstrated by pre- and post-test assessments in a pilot with 30 internal medicine residents [14]. It is free to implement using the published protocol. Still, its major limitations are that it requires significant preparation time, is not a retail product, and requires dedicated group session time.

The fourth-ranked game is Epipocalypse: Bug Detectives, also published by Lab Beagle Games [15]. Players draft scientist cards and search a shared play area to collect infection cards that correspond to their scientist cards by disease type, geographic distribution, reservoir, or vector. It supports competitive play and a solo campaign mode. Its learning objectives are to master the presentation and epidemiology of over 100 real infectious diseases and to build interleaved recall skills useful for board examination preparation [16,17]. It is currently free as a print-and-play prototype [18]. Still, its main limitations are a lack of retail polish, the need for do-it-yourself assembly, and the absence of published studies on educational efficacy.

The fifth-ranked game is Healing Blade: Defenders of Soma (Second Edition), published by Nerdcore Medical [19]. This is an asymmetric two-player card-battle game pitting antibiotic heroes against bacterial monsters, where one player attacks non-player-character villagers with disease cards and the other defends using antibiotic cards that reflect the real spectra of activity and resistance mechanisms. Its learning objectives are to understand antibiotic classes, grasp the consequences of selective pressure, and build familiarity with pathogen names. It retails for approximately $60-$70 and is widely available [19-21]. While its strengths are accurate spectrum modeling and high-quality fantasy artwork, its limitations include a high price, a two-player limit, and a fantasy abstraction that requires translation back into real clinical practice. Ranking the five games specifically for medical students, pharmacy students, residents, and fellows, weighting clinical relevance, depth, evidence base, and stewardship focus over general polish or entertainment value, produces the order in Table 2.

**Table 2:** Ranking rationale: best-to-worst fit for medical learners IDSA: Infectious Diseases Society of America, CDC: Centers for Disease Control and Prevention, AAP: American Academy of Pediatrics, CME: continuing medical education

Rank	Game	Key rationale
1	Empiric	Directly built on IDSA/CDC/AAP guidelines; scores down guideline-discordant choices; validated at CME conferences with residents and clinicians.
2	Pharmageddon: Bugs vs Drugs	Combines pathogen-drug matching with explicit stewardship trade-offs (side effects, flora damage, resistance); solo/co-op modes suit self-study.
3	Pathogen Pursuit	Real clinical organisms and treatments; only game here with published pre/post knowledge-gain data, but requires a facilitator and has a small evidence base.
4	Epipocalypse: Bug Detectives	Broadest disease/epidemiology coverage (100+ diseases), strong for ID/travel-medicine review, but unfinished product with no efficacy data.
5	Healing Blade: Defenders of Soma	Accurate antibiotic spectra and resistance modeling, but fantasy abstraction, two-player limit, and high price reduce fit for group/self-study use.

The evaluation also considered two expansion packs for Pharmageddon: Bugs vs Drugs from Lab Beagle Games. The first, Pharmageddon: ID Consult [22], is a 35-card expansion introducing a source-control mechanic in which certain infections require surgical or procedural intervention to resolve, adding clinical depth for medical trainees. The second, Pharmageddon: Top Secret [23], is a smaller, 17- to 18-card pack of whimsical wild cards that adds novelty but offers minimal educational value. Neither expansion alters the base game's overall second-place ranking, though the source-control mechanic in ID Consult enhances its clinical realism.

## Discussion

Our qualitative ranking highlights key observations about the suitability of these games for medical, pharmacy, and graduate clinical trainees. Empiric achieves the top rank because it directly scores players down for guideline-discordant choices, reinforcing the nuanced prescribing judgment required in clinical practice rather than simple rote memorization. It has been validated in continuing medical education and residency contexts, and its free accessibility makes it highly accessible. Pharmageddon ranks second because it balances pathogen-drug matching against the physiological and clinical trade-offs of antibiotic overuse, including side effects, resistance, and microbiome disruption. Its inclusion of robust solo and cooperative modes makes it the most practical tool on the list for independent, self-paced board exam preparation.

Pathogen Pursuit ranks third primarily because of its format. While it is the only game on this list with direct, published pre- and post-test knowledge-gain data demonstrating its educational efficacy in residency training [15,16], it is designed strictly as a facilitated group protocol. This design prevents independent self-study and requires significant academic preparation and schedule coordination, making it less practical than the top two selections. Epipocalypse offers the broadest clinical scope, with over 100 diseases, which is excellent for infectious disease and travel medicine board examination review. Still, it ranks fourth because it remains an unpolished, do-it-yourself prototype without published efficacy data. Healing Blade ranks last because its fantasy wrapper, while highly engaging, introduces an unnecessary translation step between clinical reality and gameplay, and its high cost and two-player limit restrict its utility for large-scale institutional use or solo study.

Several cross-cutting observations emerge from this analysis. First, higher retail cost and production polish do not correlate with greater educational utility for clinical learners, as evidenced by the superior clinical design of free and low-cost titles such as Empiric and Pharmageddon. Second, the availability of a solo-play mode heavily influences a game's practicality for individual board preparation, making self-paced games more useful for medical students and residents than group-dependent protocols. Third, the evidence base supporting these games is highly uneven: academic games demonstrate empirically validated learning outcomes, whereas commercial titles rely primarily on design credentials and user reviews. Finally, games that actively penalize inappropriate broad-spectrum prescribing align far better with modern antimicrobial stewardship priorities than games that simply reward basic pathogen-drug matching.

This evaluation has important limitations. Rankings reflect the qualitative judgment of the report's authors rather than a standardized or validated scoring instrument, and most rankings rest on expert opinion rather than direct, controlled comparison of educational effectiveness; only Pathogen Pursuit and Empiric have published outcome data, and no game has been evaluated against another in a head-to-head study. The search strategy was similarly exploratory rather than systematic, so readers seeking a fully reproducible literature search or a PRISMA-style accounting of records identified and excluded will not find one here. The rankings should therefore be read as informed guidance for selecting among currently available games, not as definitive comparative evidence.

## Conclusions

Based on a structured evaluation of clinical guidelines, stewardship principles, evidence of learning benefit, and educational practicality, the five games are qualitatively ranked from most to least suitable: Empiric, Pharmageddon, Pathogen Pursuit, Epipocalypse, and Healing Blade. In the authors' qualitative judgment, Empiric and Pharmageddon represent the strongest and most versatile choices for medical students, pharmacy students, residents, and fellows seeking to independently reinforce prescribing guidelines and stewardship concepts. This assessment is informed by Empiric's guideline-aligned scoring and educational validation, as well as Pharmageddon's focus on stewardship trade-offs and solo-play capabilities; it should be understood as an expert-informed narrative ranking rather than a finding of demonstrated comparative educational effectiveness, since most games, including these two, have not been directly compared against one another in controlled studies.

While Pathogen Pursuit remains highly effective for structured, facilitated classroom sessions due to its proven educational efficacy, its requirement for academic prep time and group scheduling limits its utility for individual study. Both Epipocalypse and Healing Blade, while possessing unique strengths in breadth of disease coverage and thematic engagement, respectively, are held back by practical limitations such as prototype status and fantasy abstraction. Ultimately, clinical training programs and individual learners should prioritize free, guideline-derived, and stewardship-focused games to maximize educational impact.
